# Killing me Softly? Scrutinising the Role of Soft Law in Greece’s Response to COVID-19

**DOI:** 10.1017/err.2020.114

**Published:** 2021-03

**Authors:** Evangelia (Lilian) TSOURDI, Niovi VAVOULA

**Affiliations:** * Assistant Professor and Dutch Research Council (NWO) grantee, University of Maastricht, Faculty of Law and Maastricht Centre for European Law, Maastricht, The Netherlands; email: e.tsourdi@maastrichtuniversity.nl; ** Lecturer in Migration and Security, Queen Mary University of London, School of Law, London, UK.

## Abstract

Greece emerged as the EU’s poster child in the fight against COVID-19 during the first few months of the pandemic. In this contribution, we assess Greece’s use of soft regulation in its regulatory response to COVID-19. Using “acts of legislative content”, which can be broadly conceptualised as softly adopted hard law, the Greek government largely achieved flexibility and simplified adoption procedures without having to resort to soft law per se. The role of soft law was limited - it complemented hard law rather than constituting the primary basis of COVID-19 restrictions - but not completely negligible. Soft law instruments regulated the processing of personal data, and was also pivotal in clarifying the criminal sanctioning of COVID-related rule violations. Greece’s success in handling the first wave of the pandemic, while effective, was arguably unfair to asylum seekers who saw their right to apply for asylum curtailed, and their right to freedom of movement restricted when limitations on the rest of the population were lifted. With a second wave of infections currently in full swing, it is imperative to keep scrutinising regulatory responses to ensure that they place the health and dignity of every individual (whoever they might be) at their core and fully respect their fundamental rights.

## I. Introduction

Greece emerged as the EU’s poster child in the fight against COVID-19 during the first months of the pandemic. Despite the country’s weakened economy and public health system, Greece was extremely effective in addressing health-related risks linked with the spread of the coronavirus. Adopting a proactive stance, in March 2020 the country introduced significant limitations to, among other things, freedom of movement, the right to private life, freedom of religion and freedom of assembly. Having secured extremely low infection rates and COVID-19-related deaths, the country gradually started lifting lockdown measures and relevant restrictions in the course of May 2020. The loosening of the initial measures culminated in late June and early July 2020, also fuelled by Greece’s desire to stimulate the national economy through an active tourist season. Nonetheless, a differentiated approach and exemptions persisted when it came to lifting restrictions. A particularly pronounced exemption was seen in respect of asylum seekers.

The effectiveness of the measures does not place them beyond scrutiny. In this contribution, we provide a holistic and nuanced assessment of Greece’s regulatory response to the pandemic, focusing on the role of soft law. In this respect, we first position soft regulation within the broader architecture of COVID-19-related regulation, and critically assess recourse to soft law. Given the breadth of regulatory responses to COVID-19, we then focus our assessment in four areas: restrictions on the freedom of movement; the processing of personal data in order to track movements and locate suspected patients; the right to asylum; and the recourse to criminal sanctions for violations of COVID-19-related legislation. Focusing on specific areas of regulation is a conscious choice of research design. On the one hand, it is impossible to analyse in-depth Greece’s entire regulatory response in an article. On the other hand, the specific areas of regulation chosen pose heightened challenges to fundamental rights for two reasons. First, the use of newly introduced techniques, such as patient tracking, or permits of movement issued via text message (SMS), stirs up novel, under-explored, fundamental rights challenges. Next, certain areas of regulation pose significant implications for the protection of either absolute fundamental rights, such as the prohibition of inhuman or degrading treatment, or fundamental rights that, even if not absolute, are inalienable and a *sine qua non* to exercising a number of other rights, such as the right to liberty and security. We examine whether soft law has been used merely to complement legislative measures and administrative rule-making, or whether it independently formed the basis for the adoption of restrictions. We analyse the constitutionality of soft law measures and their adherence to EU, European and international human rights standards, by reflecting on their necessity and proportionality. We conclude with critical remarks on Greece’s regulatory approach and its use of soft law, as well as related perspectives in view of the increasing risks COVID-19 poses to Greek society.

Researching regulatory responses to COVID-19 is an endlessly moving target; our analysis is limited to measures adopted in the period March–July 2020. The temporal proximity to events studied makes this piece one that is illustrative of “action research”. As a piece of action research, our contribution is informed by the study of primary legal sources (mainly Greek national legislation and guidelines), combined with information on its application available through governmental sources (Eg government press releases), reports of national civil society organisations (Eg Medicines Sans Frontières), European Union reports (Eg the Fundamental Rights Agency) and a wealth of journalistic sources. As noted by Alberto Alemanno, this type of research understandably carries “its own epistemic risk – the risk of error versus the risk of missing the truth”.^1^ While acknowledging the limitations of this scientific approach, we are proponents of a timely scientific scrutiny of states’ regulatory architecture in responding to COVID-19, bearing in mind its impact on individuals’ fundamental rights, including the state of exceptionality facing particular societal groups, and the enduring nature of the pandemic.

## II. The place of soft law in Greece’s regulatory architecture

To properly assess the use of soft law, it should be placed against the backdrop of the entire regulatory response that Greece deployed. The Greek Constitution does not provide for a state of emergency. Instead, it establishes the notion of an “act of legislative content” (πράξη νομοθετικού περιεχομένου).^2^ According to the Greek Constitution:

[u]nder extraordinary circumstances of an urgent and unforeseeable need, the President of the Republic may, upon the proposal of the Cabinet, issue acts of legislative content. Such acts shall be submitted to Parliament for ratification […] within forty days of their issuance or within forty days from the convocation of a parliamentary session.

Benefiting from this prescription, the Greek government adopted general measures in response to the COVID-19 outbreak in the form of “acts of legislative content”. Namely, during the period of coverage of this article, ie March–July 2020, the government adopted seven acts of legislative content, with the latest one adopted at the beginning of May 2020.^3^ All of them were subsequently ratified by Parliament. Based on these acts, the Greek executive has issued numerous (joint) ministerial decisions (κοινές ΥποΥργικές αποφάσεις, KYA).^4^ These are legally binding acts of the executive. They have touched upon various aspects of individuals’ personal and professional lives ranging from conditions for working at home and restrictions in free movement to measures of financial support for different categories of workers. The constitutionality of resorting to acts of legislative content, and subsequently to ministerial decisions, to adopt such far-reaching restrictions has been characterised as “dubious”.^5^ This is, however, a matter that goes beyond the scope of the present contribution.

Soft law enters the regulatory scene first in the form of interpretative circulars (ερμηνεΥτικές εγκύκλιοι).^6^ Interpretative circulars are issued to add further explanatory content to the provisions in the acts of legislative content as well as to the more precise provisions of the (joint) ministerial provisions. Under Greek administrative law, the role of circulars is central. They contain an interpretation of a piece of legislation, and are meant to harmonise and unify its implementation by administrative bodies. The underlying objective is to enhance the effectiveness of public administration and the protection of individuals’ rights. The content of circulars is binding on hierarchically lower administrative bodies. However, they are only binding on the administration and not on external third parties. A circular does not constitute an enforceable administrative act, and hence cannot be the subject of an act for annulment. The Greek Council of State, the highest administrative court in Greece, has, however, annulled acts that introduced, for the first time in the Greek legal order, binding enforceable norms falsely taking the form of a circular.^7^ In essence, a circular promotes the creation of uniform administrative practice and is directed at the inner circle of public administration aiming at the optimal organisation and operation of administrative bodies. As a result, a circular exhausts its binding effect within the remits of the administration.

Apart from circulars, soft law enters the regulatory scene through so-called “guidelines” (οδηγίες). In practice, they are often issued by independent authorities, such as the Hellenic Data Protection Authority in our case. These administrative bodies use guidelines to curb their own discretion where discretion is provided by law.^8^ Thus, guidelines contribute to the rationalisation and effectiveness of administrative action. In addition, they protect individuals as they enhance a uniform application of administrative discretion and avoid unjustified differentiation in similar situations.^9^ Finally, while not justiciable themselves, they simplify judicial control of the legality of individual administrative acts, as the administration should abide by the limits that it has set itself as to its discretion.^10^

## III. Greece’s use of soft law to combat COVID-19 under scrutiny

In the following sections, we examine Greece’s response to the pandemic and its use of soft law focusing on four areas: establishing restrictions to freedom of movement; privacy and data protection; the right to asylum; and criminal sanctions. We assess the importance of the relevant soft law and its relationship with national binding legislation adopted in these areas, as well as the compatibility of that soft law with EU and international law.

### 1. Restrictions to the freedom of movement: a case study in “othering”?

A prime way to contain the spread of the virus and protect public health has been the imposition of physical distancing through temporary lockdown and stay-at-home requirements. We examine first the role of soft law in generally applicable measures. We then focus on asylum seekers. This population group was subjected to a set of enhanced restrictions, which persisted even as restrictions for the general population were eased.

#### a. Generally applicable measures: a balanced approach

The Greek government issued a ban on all unnecessary traffic within Greek territory from 22 March 2020 to stop the spread of COVID-19, which lasted initially until 6 April 2020, and was later continuously prolonged until 4 May 2020.^11^ Certain exhaustively defined exceptions applied to essential movements, which included buying food and medicine, attending medical appointments, going to work, training outdoors alone or with one other person, or walking a pet. Anyone on the move falling within one of the expressly listed exceptions was required to carry a police-issued identity card or passport as well as a permit of movement.^12^ This permit could be obtained by filling out an online form, or a paper form listing the reason for leaving home. By far the most popular option though, was to send an SMS via a mobile phone to a dedicated number operated by the General Secretariat of Civil Protection (Γενική Γραμματεία Πολιτικής Προστασίας). We examine these measures through a data protection and privacy perspective below.^13^ As the epidemiological situation in Greece improved, stay-at-home orders were lifted and a special permit was no longer needed as of 4 May 2020. However, movement was restricted to the district of residence for everyone until 18 May 2020.^14^ After that date, restrictions to the freedom of movement for the general population were lifted, and the public was then encouraged to use personal protection measures. Social distancing measures, such as the use of face masks, were introduced.

These measures were established through hard law; namely via a series of joint ministerial decisions. To our knowledge, soft law was not used in relation to restricting the movements of the general population. Rather it was used in clarifying social distancing measures, such as the use of face masks,^15^ or in establishing the number of passengers in private cars.^16^ Most of these measures followed the lifting of restrictions. However, we consider that these social distancing measures were far less invasive than restrictions to freedom of movement and thus less likely to seriously affect fundamental rights. Hence, they do not form a dedicated subject of our analysis. As for the legislative measures, they certainly affected the right to freedom of movement and residence provided for in the Charter.^17^ However, given their time-bound nature, their active review, and their prompt lifting based on objective indicators, such as the epidemiological situation in the country, our view is that they were necessary and proportional.^18^ The same picture does not emerge when examining the situation of asylum seekers.

#### b. The special position of asylum seekers: unjustified restrictions?

A different position altogether was adopted regarding restrictions on the freedom of movement of asylum seekers. On 17 March, the General Secretariat for Reception of Asylum Seekers of the Ministry of Immigration and Asylum issued several guidelines (οδηγιες), the so-called “Agnodiki Plan”, that were addressed to all asylum reception centres, as well as reception and identification centres, in Greece. Information on the content of these guidelines is accessible through a press release.^19^ The guidelines set out different levels of COVID response. The legal nature of these guidelines is not clear; the press release mentions that they were issued “on the basis of the official instructions of the Greek Government”, obviously referring to the relevant acts of legislative content.^20^ Given the nature of the measures outlined in the press release, and its wording, we conclude that these “guidelines” constitute soft law. They established far-reaching restrictions, including the suspension of any recreational activities and schooling. They also limited the movements of asylum seekers both outside and within these centres to those strictly necessary.

Upholding these far-reaching limitations, which deviated from the restrictions applicable to the general population, through guidelines alone would have been constitutionally dubious. These initial soft law guidelines were, however, swiftly followed up by legally binding measures. Ever since, restrictions have been specified and concretely based on legally binding measures. However, the soft law Agnodiki Plan is the document that sets out the overall operational philosophy and, hence, underpins these legally binding documents. On 21 March 2020, a Joint Ministerial Decision was issued crystallising far-reaching restrictions to the freedom of movement of asylum seekers in reception and identification centres, initially for a one-month duration.^21^ These limitations included the possibility of circulating outside the centres, but only between 07.00 and 19.00 hrs, and only for the purpose of covering “basic needs”. In addition, the decision abolished any activities in the centres and camps other than those related to housing, subsistence and healthcare provision. These restrictions were repeatedly renewed during the period we are concerned with, through joint ministerial decisions: first until 10 May 2020,^22^ then until 21 May 2020,^23^ thereafter until 7 June 2020,^24^ 21 June 2020,^25^ 5 July 2020,^26^ 19 July 2020,^27^ and finally 2 August 2020.^28^ Moreover, apart from these generally applicable restrictions, even tighter restrictions were applied in specific centres based on joint ministerial decisions.^29^ The restrictions remained in place at the time of writing, ie throughout September 2020. This means that long after restrictions based on public health considerations had been eased for the general population, far-reaching restrictions persisted for asylum seekers.

Beyond these restrictions mandated by law, further restrictions were applied *de facto* to those newly arrived on the island of Lesvos as of 22 March 2020. Namely, there are reports of persons placed under restriction of freedom of movement outside the reception and identification centres, in specifically designated areas of the island countryside such as Megala Therma, offering no shelter from extreme weather conditions, and no access to toilets, showers or running water.^30^ Newly arrived asylum seekers remained in such conditions for several weeks in some cases. On 28 May 2020, pursuant to a complaint submitted by HIAS Greece, the Greek Ombudsman intervened, requesting information about, among others, the rationale for the non-placement of new arrivals in appropriate quarantine areas and for the extension of the quarantine beyond 14 days.^31^ Nevertheless, there remained reports of quarantining newcomers in substandard conditions in July 2020.^32^

Apart from the initial guidelines (which were first issued as soft law and then as legislative measures), soft law in the form of a circular was used to clarify measures and conditions in asylum centres for unaccompanied minors.^33^ The circular included instructions regarding personal hygiene measures for the children and staff, instructions on cleaning and disinfection, and detailed instructions in case of illness. Given its content and its wording this was a typical interpretative circular. We assume that special attention was afforded to centres hosting unaccompanied asylum-seeking minors given the heightened vulnerability of this population group.^34^

There are several legal concerns with the approach Greece took regarding asylum seekers. First, the conditions in several of these centres arguably violate the principles of human dignity and the prohibition of inhuman or degrading treatment.^35^ To illustrate, asylum seekers on the islands’ “hotspots”,^36^ where many are accommodated, face unsanitary conditions. There was only one shower for every 500 people, and one toilet for every 160, and meal services were congested.^37^ This led the European Commission,^38^ Members of the European Parliament,^39^ numerous civil society organisations,^40^ and the EU’s Fundamental Rights Agency,^41^ among others, to call for the evacuation of refugee camps in Greece and for the activation of intra-EU solidarity measures. They were also joined by the European Court of Human Rights, which ordered interim measures in the case of *EI and others v Greece*,^42^ requiring that several people be moved immediately out of Moria, a “hotspot” functioning in Lesbos, to obtain medical treatment and to ensure that they were not exposed to a risk of inhuman or degrading treatment. Despite these calls, neither Greece nor the EU facilitated a mass evacuation of these camps.^43^

The second observation relates to restrictions on the freedom of movement of asylum seekers. These restrictions were not only incompatible with fundamental rights due to the living conditions in camps, but were more intense and lasted longer compared to those applicable to the general population. According to international human rights law,^44^ while freedom of movement can be restricted for reasons of public health these restrictions should also comply with the principle of necessity.^45^ With the easing of restrictions for the general population based on the improved epidemiological situation in the country, it is not clear whether this condition was fulfilled, and on what basis the risk posed by this specific group was assessed. If anything, immobilising asylum seekers in squalid conditions where social distancing and other hygiene measures were rendered almost impossible arguably jeopardised both public health and their own right to health.

### 2. (Increased) processing of personal data, privacy implications and the role of guidelines

Restrictions on freedom of movement go hand in hand with efforts to monitor those on the move, as well as their personal associations in case they are infected. This section will focus on the role of soft law in information processed when people obtained permits of movement via SMS, as well as that applicable to patient tracking. Emphasis in this context is placed on the Hellenic Data Protection Authority (DPA) in interpreting legislation through guidelines.

#### a. Permits of movement and their regulation through data protection (soft) law

As already discussed, the acquisition via SMS was the most frequently used option for securing a permit of movement. In obtaining permission via SMS, individuals were required to state their name and surname, list the code number of the purpose of movement and provide their residential address. In the event of a random check by law enforcement authorities, individuals were required to demonstrate their permit, otherwise a fine would be imposed.^46^ A key question in this context is the extent to which the Greek government collected and processed *en masse* personal data provided by individuals who opted for obtaining a permit by sending an SMS.

The rules on the processing of this personal data were not adopted via a ministerial decision. Instead, the General Secretariat for Civil Protection, a public law body that organically belongs to the Ministry of Citizen Protection, merely released online a “data protection policy”, in the form of soft law, without prior scrutiny, consultation or transparency.^47^ The government opted for this despite its possible implications for the legality of data processing and the impact on individuals whose information was processed. The data protection policy stressed that the data were not retained by the Greek authorities and would be deleted after use. Therefore, after the citizen received the SMS message with the exit permit, the data would be erased or anonymised.

One could argue that, in view of the limited time frame to which the measure applied and the deletion of the data after the issuance of the permit, there was no need for further formalisation of the rules. Perhaps this explains why the data protection policy in relation to the permits has been subsequently deleted and can no longer be located online. However, whereas no centralised storage was foreseen, the policy attracted criticism^48^ for its use of legal language that was inaccessible to the public, for the absence of any reference to the fact that it could be used to collect sensitive data^49^ and, most importantly, for the confusion as to whether the information submitted by individuals could be transferred to third parties.

#### b. Proportionality concerns with the tracking of COVID-19 patients

This analysis showcases how technological means have been a crucial component in efforts to contain the spread of the virus and protect public health, raising significant privacy and data protection concerns.^50^ Indeed, nowhere has the evolution of technology been more relevant in responding to COVID-19 than in so-called “exit strategies”, particularly apps and other tools to trace and track the contacts of persons suspected of or diagnosed with COVID-19. At the time of writing, the Greek government is still in the process of evaluating the different application models that have been proposed in the past months,^51^ and so an appraisal of the relevant legislative and soft law framework is impossible.

In the meantime, contact tracing takes place through traditional means of collection of patient data. Such collection has been mandated by acts of legislative content. In particular, Article 5 of the Act of Legislative Content of 14 March 2020 mandates the collection of personal data of potentially or actually infected persons by the Hellenic National Public Health Organisation (εθνικός Oργανισμός Δημόσιας Υγείας, εOΔΥ), a private law entity, with the aim of sharing it with the General Secretariat for Civil Protection.^52^ The data shared include the name, gender, age, contact number and full address, as well as information on whether the individual has been hospitalised and if so, in which hospital and, where relevant, the place of self-isolation.^53^ The data are pseudo-anonymised and their transmission encrypted;^54^ and the processing of the data is limited to the purposes of coordination between the Hellenic National Public Health Organisation and the General Secretariat for Civil Protection for the effective fight of COVID-19.

In terms of the period for which data are retained, the Act foresees the storage of collected data throughout the duration of the urgent measures, but not after the end of 2020.^55^ In addition, Article 29 of the Act of Legislative Content of 30 March 2020 established a National Registry of COVID-19 patients, which regulates the processing of personal data and individual rights.^56^ The Ministry of Health issued a Ministerial Decision on 14 April 2020 for the implementation of that registry. According to the decision 2650/10.04.2020 of the Ministers of Health and Digital Governance that was issued later, the data are to be kept almost indefinitely, as they can be kept for further 20 years after the individual’s death.^57^ The lack of constitutionality of this provision is striking.^58^ In a series of judgments, both the Court of Justice of the European Union (CJEU) and the European Court of Human Rights (ECtHR) have clarified that the temporal character of retention is an important element for the proportionality test. In *S and Marper v the United Kingdom,* the ECtHR emphasised that the indefinite retention of sensitive personal data, irrespective of their further use, may have a direct impact on the applicants’ private life interest, including their stigmatisation.^59^ Furthermore, in *Digital Rights Ireland*, the CJEU opined that the retention period must be based on objective criteria in order to ensure that it is limited to what is strictly necessary.^60^ In the present case, the long retention period is equated with indefinite retention, which, in line with the relevant case law, is arguably disproportionate. This is all the more the case considering that the retained data include information on the health of individuals, which qualifies as a special category of personal data in accordance with EU’s General Data Protection Regulation (GDPR).^61^

Even though the provision was introduced through a Joint Ministerial Decision, thus without recourse to soft law, it is worth noting its lack of proportionality, which is in line with the overall restrictive nature of measures adopted by the Greek government that affect privacy and data protection rights. As for the contact tracing of individuals, this process is carried out by a designated centre situated in the police headquarters. The process involves asking questions regarding the recent contacts of persons infected, or suspected of being infected, with coronavirus. In order for public bodies (particularly hospitals and clinics) to assess who constitutes a close contact and therefore must be subject to a specific set of instructions due to the high risk of contracting COVID-19, the Hellenic National Public Health Organisation (EODY) has circulated detailed guidelines specifying the relevant criteria.^62^ These guidelines have also been made public on the dedicated website of EODY, without their elaboration in an administrative act.

#### c. The guidance of the Hellenic Data Protection Authority: soft law with far-reaching implications

The use of guidelines for public authorities is further exemplified by the issuance on 18 March 2020 by the Hellenic DPA of guidelines concerning the processing of personal data within the framework of management of COVID-19, particularly the applicability of the GDPR.^63^ The DPA is an independent authority entrusted with various tasks in accordance with Articles 51–59 of the GDPR, including the issuance of opinions on its own initiative or, on request, on any issue related to the protection of personal data.^64^ To those ends, from time to time the DPA issues soft law in the form of guidelines suggesting solutions on various problems arising from the advancement of new technologies.^65^ In this context, the DPA COVID-19 guidelines focus on the use of personal data including health data by both public and private bodies especially in the field of employment and in relation to media reporting and coverage. The DPA provided a definition of health-related data that includes naming or identifying a data subject as a patient or not, whether they are staying at home due to illness, and any signs of illness based on their clinical symptoms (cough, nasal discharge, body temperature higher than normal, etc).^66^ According to the DPA, such information falls within the realms of the GDPR only when processed wholly or partly by automated means, and not when provided orally.^67^ Therefore, the DPA guidelines are far from technical in nature and provide an interpretation of the GDPR in numerous respects.

Furthermore, the DPA rightly states a series of applicable legal bases for the processing of personal data for COVID-19 related purposes,^68^ provided that the basic principles are met and that relevant substantive and procedural safeguards and conditions for lawful processing are ensured.^69^ The DPA further emphasises the processing of personal data by the private sector in the framework of employment relationships. It opines that, insofar as the GDPR applies, employers are entitled to process personal data in order to protect the health of employees. Practices such as measuring the body temperature of incoming individuals, submitting questionnaires regarding the health status of employees or their relatives and their travel history, or informing other employees of the fact that a fellow employee has been infected and of the employee’s identity, are explicitly allowed.

It is noteworthy that in view of the “critical and unprecedented time”, the DPA stresses that no policy choice could be outright excluded from scrutiny. However, the key data protection principles, as enshrined in Article 5 and 6 of the GDPR, are applicable. Thus, the DPA rightly notes that the extensive collection of personal data resulting in profiling of employees does not comply with the principle of proportionality.^70^ As Evelina Pappa points out, the guidelines are not particularly clear, and in comparison to guidelines provided by national DPAs in other EU Member States, the position is somewhat overly permissive.^71^ As a result, the view of the DPA seems to be informed by the state of emergency and, if implemented by the administration, may have a considerable impact on the rights to respect for private life and to protection of personal data. Finally, the transfer of information relating to the health status of individuals is prohibited “where it is creating a climate of prejudice and stigma, while it is also likely to have a preventive effect with regard to complying with the measures announced by the competent public authorities undermining eventually their effectiveness”.^72^

### 3. Right to asylum: the role of the pandemic in a continuum of restrictions

The right to asylum was suspended in Greece about two weeks prior to the adoption of any COVID-19-related restrictions. Following a spat with Turkey at its borders, Greece, through an act of legislative content unrelated to COVID-19, suspended for the entire month of March 2020 the right to asylum for those newly arrived and stipulated immediate deportation of those entering Greek territory.^73^ That legal act formed part of the Greek response to Turkey’s declaration that it would no longer prevent refugees and migrants from crossing to Greece. The suspension arguably violated international and EU law – a topic that goes beyond the scope of the present contribution.^74^ The suspension had highly damaging effects on a significant number of people in need of protection, with thousands arbitrarily detained without effective judicial protection.^75^ Under international and EU law, asylum seekers cannot be automatically detained. They can only be deprived of their liberty if one of six exhaustively enumerated detention grounds applies, and based on an individualised assessment.^76^

The breakout of the pandemic led to a continuum of restrictions relating to the right to asylum. Namely, a Joint Ministerial Decision of the Minister of Health and the Minister of Interior, Migration and Asylum called for the closure to the public of the offices of the Asylum Service in Athens and other regional offices between 12 March and 10 April 2020.^77^ This was later extended to 15 May 2020.^78^ During this period, no registration of new asylum applications was possible, no interviews took place and no appeals were registered.^79^ The Asylum Service resumed its services only on 18 May 2020.

This meant that the two layers of restrictions coincided during March 2020, while only the COVID-19-related restrictions stood thereafter. Thus, through a seamless web of restrictions, asylum seekers arriving from 1 March 2020 onwards had no access to asylum for more than two and a half months. Nonetheless, protection needs cannot be set aside, either in the exercise of border controls or while implementing measures to address public health considerations.^80^ In addition, as good practice collected by the UNHCR illustrates, it is possible to ensure public health and maintain basic registration and documentation functions, as well as preventing backlogs.^81^

Asylum seekers also faced a continuum of restrictions regarding their right to liberty and security, and to their right to freedom of movement. The Greek policy of blanket detention of all new arrivals on the basis of the initial “immediate deportation decree” was followed by the arguably disproportionate restrictions to their freedom of movement, discussed above.^82^ An unfortunate conclusion is that the pandemic served partly as a fig leaf to consolidate restrictions to the right to asylum and the right to freedom of movement based on completely different considerations. Similar restrictive trends to the right to asylum under the guise of COVID-19 were reported in Cyprus and Hungary.^83^

What is the role of soft law in all this? This area allows us to develop interesting observations regarding the outer boundaries of what constitutes soft law in Greek administrative law. As already analysed, the legal basis of these measures was “hard law”: acts of legislative content and joint ministerial decisions. There were also some additional documents issued by Greek administrative authorities. Namely, on 11 March 2020 the Migration Policy Service of the Ministry of Migration and Asylum issued a document titled “Suspension of receiving and servicing the public” directed to several other Ministries and administrative services.^84^ A similar document was issued by the same service regarding the extension of the suspension on 8 April 2020.^85^

Studying the content and the wording of these documents we conclude that they do not constitute soft law. They merely repeat the content of the ministerial decisions, without however providing any further clarifications or a specific interpretation of the relevant provisions. They also offer no indications as to how discretion should be exercised in an area where the administration enjoys a margin of it. Therefore, we conclude that these documents are merely “announcements”. Their aim is to highlight and bring to the attention of the administration legislation that was urgently adopted. This, according to our view, brings these documents outside the realm of soft law, as if they were devoid of legal nature.

### 4. The pivotal role of soft law in implementing criminal sanctioning for violations of COVID-19 related measures

The adoption of measures constraining freedom of movement goes hand in hand with sanctions for those who do not abide by the measures to contain the spread of coronavirus. In relation to COVID-19, two types of sanctions can be discerned, both of which have a preventive function. Administrative fines of €150 may be imposed in accordance with Joint Ministerial Decision 24112/9.4.2020,^86^ which modified Joint Ministerial Decision 20036/22.3.2020 by the Ministers of Citizens’ Protection, Health and Interior.^87^ No use of soft law occurred in this context. Moreover, criminal law sanctions are prescribed in Article 285 of the Greek Penal Code, as revised by Law No 4619/2019, which came into effect on 1 July 2019, less than a year before the pandemic.

In what could be described as a prophetic move because the enactment of Article 385 took place before the pandemic, the Greek Penal Code revamped the criminal offence of violating measures for the prevention of diseases^88^ and Article 285 foresees a crime of danger,^89^ and prescribes a gradation of penalties from pecuniary sanctions to a custodial sentence. In particular, if the disease is transmitted to an indeterminate number of people on purpose, the offence is punishable by a fine and imprisonment for up to five years. In addition, the *mens rea* for that offence may be negligence, in which case the offence is punishable with a custodial sentence of up to two years or a financial penalty due to its limited wrongfulness.^90^ An aggravated form of the offence is prescribed in paragraph 3, according to which if the violation results in the death of a person, or a large number of persons, then the imposed custodial sentence may range from at least ten years to life imprisonment.

Just a few months following its adoption, the need to implement this Article has come to the fore with the role of the prosecutorial authorities becoming pivotal in this respect. Indeed, the Office of the Public Prosecutor at the Court of Cassation in Greece issued two circular orders in March 2020 which are worth noting. Although prosecutorial authorities –at least in Greece– are considered to be independent from the executive authority, it is notable that no other circulars of legal value have been issued in connection to the Ministry of Justice, apart from those issued by the office of the Public Prosecutor.^91^

Circular order no 4/12.3.2020^92^ informed the prosecutors’ offices at all First Instance Courts of Greece, as well as the prosecutors’ offices at the Greek Courts of Appeal, of the content of Article 285, which imposes much increased penalties on those who endanger legal interests (life, health, etc). In the following circular no 7/31.3.2020,^93^ the Office of the Public Prosecutor at the Court of Cassation in Greece had a more alarming tone, referring to the immediate need for “vigilance and constant intervention” for the prosecution of crimes relating to the COVID-19 pandemic. This includes the relevant crimes of the Greek Penal Code, such as the violation of measures for the prevention of diseases (Article 285), usury (Article 404), blackmail, etc. The reference to vigilance is crucial in this context and sets the tone for national implementation.

However, the constitutionality of Article 285 merits further scrutiny, in view of Article 5(3) of the Greek Constitution, which enshrines the right to personal liberty. Furthermore, in light of Article 25(1) of the Constitution, individual rights and the rule of law are guaranteed by the state, and restrictions to constitutional rights may be imposed by the constitution or the law in accordance with the principle of proportionality. In addition, the Greek Constitution holds that the state cares for the health of citizens,^94^ and has the right to claim that all citizens should fulfil the duty of social and national solidarity.^95^ However, the severe criminal sanctions (when the offence is classified as either a misdemeanour or a felony), as outlined above, coupled with the evidentiary difficulties in proving the concrete danger of the transmission of COVID-19 posed by the violation of measures, herald a cautious application of Article 285.^96^ Indeed, despite the tone of the circular orders, and to the best of our knowledge, the imposition of administrative fines was favoured during the research period over the initiation of criminal proceedings, at least in the first wave of the pandemic.^97^ As a result, criminal proceedings have been rightly reserved only for the most serious cases, for example in connection with violations by a private clinic where 35 COVID-19 cases occurred in April 2020 resulting in the death of 13 patients.^98^

## IV. Greece and COVID-19: regulatory perspectives

It has been argued that the primary advantage of soft law in times of crisis is its flexibility, with simplified soft law adoption procedures being highly valued characteristics in addressing situations where swift action is imperative.^99^ Through the vehicle of acts of legislative content, the Greek government largely achieved the goal of flexibility and simplified adoption procedures without having to resort to soft law, at least not as heavily perhaps as other countries. Acts of legislative content can thus be largely conceptualised as softly adopted hard law.

The overall volume of COVID-19 related soft law in Greece was fairly limited in the fields scrutinised above, and it largely complemented hard law, rather than constituting the primary basis of restrictions. The role of soft law, which is applicable to the restrictions to freedom of movement, illustrates this. In what concerns restrictions to the right of asylum, the “announcements” issued by the Greek administration are arguably of no legal value, ie they do not constitute soft law as they provide no further clarification or refinement of the relevant hard law instruments.

This does not mean that the role of soft law was utterly negligible. While contact tracing was mandated by an act of legislative content, Hellenic DPA guidelines regulated the processing of personal data, directly impacting the rights to privacy and data protection. Circulars from the Office of the Public Prosecutor at the Court of Cassation in Greece were also pivotal in clarifying criminal sanctioning of COVID-19-related rule violations. Had they been religiously followed by prosecutors, they could arguably have interfered disproportionately with the right to personal liberty. The non-legally-binding Agnodiki Plan is the document that outlines the operational philosophy around restrictions to the freedom of movement of asylum seekers in reception and identification centres; the restrictions are then specified and implemented on the basis of legally binding instruments.

Our analysis revealed that Greece’s handling of the first wave of the pandemic, while effective, was not beyond reproach. The greatest victims were asylum seekers who saw their right to apply for asylum severely curtailed, and their right to freedom of movement arguably unjustifiably restricted when limitations on the rest of the population were lifted. Greece’s COVID-19 regulation is intrinsically linked with the tragedy in Moria, which took place in September 2020. This wildly overcrowded refugee camp in Lesbos, into which asylum seekers were placed in unsanitary conditions that jeopardised not only public health but also human dignity, burnt down to the ground leaving thousands starving and homeless.

The pandemic is far from over, with a second wave of infections currently in full swing. Given the far-reaching effects of COVID-19 regulation on fundamental rights, it is imperative to keep scrutinising regulatory responses. It is to be hoped that, whatever the regulatory modes chosen, Greece’s and the EU’s responses in the next months of the pandemic will place the health and dignity of every individual at their core, regardless of who those individuals are, and will be in full respect of their fundamental rights.

## Footnotes

1 See A Alemanno, “Taming COVID-19 By Regulation: An Opportunity for Self-Reflection” (2020) 11 European Journal of Risk Regulation 187, 187 and note 1.

2 See Greek Constitution, Art 44 <www.wipo.int/edocs/lexdocs/laws/en/gr/gr220en.pdf> (last accessed 7 October 2020).

3 See Act of Legislative Content, “Emergency measures of prevention and limitation of the contagion of the coronavirus” OG A’ 42/25-2-2020 <www.dsanet.gr/Epikairothta/Nomothesia/42_2020.htm> (last accessed 7 October 2020); Act of Legislative Content, “Emergency measures to counteract the negative impact of the coronavirus COVID-19 emergence and the need to limit its contagion” OG A’ 55/11-3-2020 <www.dsanet.gr/Epikairothta/Nomothesia/PNP_11-3-2020.htm> (last accessed 7 October 2020); Act of Legislative content, “Emergency measures in response to the need to limit the dispersion of the coronavirus COVID-19” OG A’ 64/14-3-2020 <www.dsanet.gr/Epikairothta/Nomothesia/PNP_14-3-2020.htm> (last accessed 7 October 2020). Another two followed at the end of March and mid-April. Act of Legislative Content, “Measures to tackle coronavirus COVID-19 pandemic and other urgent provisions” OG A ’75/30-3-2020 <www.dsanet.gr/Epikairothta/Nomothesia/pnp30032020.htm> (last accessed 7 October 2020); Act of Legislative Content, “Measures to tackle the continuing impact of the Coronavirus COVID-19 pandemic and other urgent provisions” OG A ’84/13-4-2020 <www.dsanet.gr/Epikairothta/Nomothesia/pnp13-4-2020.htm> (last accessed 7 October 2020). The last Act was adopted at the beginning of May. See Act of Legislative Content, “Further measures to tackle the continuing impact of the Coronavirus COVID-19 pandemic and the return to social and financial normality” OG A ’90/1-5-2020 <www.dsanet.gr/Epikairothta/Nomothesia/PNP01-05-2020.htm> (last accessed 7 October 2020).

4 For a codification of all Greek legislation that was adopted as a response to the pandemic see “COVID-19: Codification of legislation of urgent measures by the Ministry of Digital Governance” (*Lawspot*, 3 September 2020) <www.lawspot.gr/nomika-nea/covid-19-kodikopoiisi-nomothesias-katepeigonton-metron-apo-ypoyrgeio-psifiakis?fbclid=IwAR2MKhCBvCwajWK58uRLPHzU8dkc0C_mCWjiBpZ5tQ2lHRnhy9uuvu6pfd> (last accessed 7 October 2020).

5 See for analysis E Fasia, “Effective but Constitutionally Dubious: The Constitutionality of Greece’s Response to the Pandemic” (*Verfassungsblog*, 23 April 2020) <verfassungsblog.de/effective-but-constitutionally-dubious/> (last accessed 7 October 2020).

6 There are two further types of circulars in Greek administrative law: so-called orientation circulars that announce a future policy, and circulars that focus on the internal organization of an administrative service and its functioning. However, these other types are not relevant to our analysis.

7 See analysis in E Prevedourou, *Soft Law Norms in Administrative Law* (“Kανονες Soft Law Στο Διοικητικο Δικαιο”) (Antonios N Sakoulas editions 2017) para 88 referring to cases Greek Council of State 3319/2015 and Greek Council of State 2754/2012.

8 ibid, para 98.

9 See analysis in P Pavlopoulos, *La Directive en Droit Administrative* (LGDJ 1978) 94 et seq.

10 Prevedourou, supra, note 7, paras 102–3.

11 Ministries of Citizen Protection, Health and Interior, Joint Ministerial Decision, “Imposition of a measure to temporarily restrict the movement of citizens in response to the risk of spreading the COVID-19 coronavirus” No D1a/GP oik 20036, OG B’ 986/22-3-2020 <bit.ly/2Ud69we> (in Greek) (last accessed 7 October 2020).

12 Greece, Ministries of Citizen Protection-Asylum and Migration, Joint Ministerial Decision, “Extension of D1a/GP.oik 26794 (B’ 986) Joint Ministerial Decision of […] until 04.5.2020”, OJ 1585/2020.

13 See below, Section III.2.

14 Greece, Joint Ministerial Decision, Ministries of Citizen Protection-Health-Interior, “Imposition of the temporary restriction […] until 18.5.2020 at 6:00 am”, No D1a/GP.oik. 27818, OG B’ 1648//2020.

15 Greece, National Public Health Organization “COVID-19 – Guidelines for the use of mask by the public” <eody.gov.gr/covid-19-odigies-gia-ti-chrisi-maskas-apo-to-koino/> (2020, in Greek) (last accessed 7 October 2020).

16 Ministry of Transport and Infrastructure, Circular, “Supplementary measures for the transport of passengers and the avoidance and limitation of Coronavirus COVID-19”, No 21656/1115, 1 April 2020 <diavgeia.gov.gr/doc/9%ce%9A%ce%A19465%ce%A7%ce%98%ce%9E-%ce%94%ce%9F4?inline=true> (in Greek) (last accessed 7 October 2020).

17 EU Charter for Fundamental Rights, Art 45.

18 EU Charter for Fundamental Rights, Art 52(1).

19 Greece, Ministry of Migration and Asylum, “Coronavirus protection measures at Reception and Identification Centers, Accommodation facilities and the Asylum Service”, press release, 18 March 2020 <bit.ly/33JHulO> (in Greek) (last accessed 7 October 2020).

20 ibid.

21 Greece, Ministry of Citizen Protection and Ministry of Migration and Asylum, “Joint Ministerial Decision on the restriction of movement in asylum seekers’ hosting facilities”, D1a/GP.oik20030/21/3/2020, OJ 985/2020.

22 Greece, Ministry of Citizen Protection and Ministry of Migration and Asylum, Joint Ministerial Decision, “Extension of D1a/GP.oik20030/21/3/2020 (B’ 985) Joint Ministerial Decision of […] until 10.5.2020”, OJ 1472/2020.

23 Greece, Ministry of Citizen Protection and Ministry of Migration and Asylum, Joint Ministerial Decision, “Extension of D1a/GP.oik20030/21/3/2020 (B’ 985) Joint Ministerial Decision of […] until 21.5.2020”, OJ 1771/2020.

24 Greece, Ministry of Citizen Protection and Ministry of Migration and Asylum, Joint Ministerial Decision, “Extension of D1a/GP.oik20030/21/3/2020 (B’ 985) Joint Ministerial Decision of […] until 7.6.2020”, OJ 1972/2020.

25 Greece, Ministry of Citizen Protection and Ministry of Migration and Asylum, Joint Ministerial Decision, “Extension of D1a/GP.oik20030/21/3/2020 (B’ 985) Joint Ministerial Decision of […] until 21.6.2020”, OJ 2191/2020.

26 Greece, Ministry of Citizen Protection and Ministry of Migration and Asylum, Joint Ministerial Decision, “Extension of D1a/GP.oik20030/21/3/2020 (B’ 985) Joint Ministerial Decision of […] until 5.7.2020”, OJ 2453/2020.

27 Greece, Ministry of Citizen Protection and Ministry of Migration and Asylum, Joint Ministerial Decision, “Extension of D1a/GP.oik20030/21/3/2020 (B’ 985) Joint Ministerial Decision of […] until 19.7.2020”, OJ 2497/2020.

28 Greece, Ministry of Citizen Protection and Ministry of Migration and Asylum, Joint Ministerial Decision, “Extension of D1a/GP.oik20030/21/3/2020 (B’ 985) Joint Ministerial Decision of […] until 2.8.2020”, OJ 2730/2020.

29 Greece, Minister of Citizen Protection, Minster of Health and Minister of Migration and Asylum, “Joint Ministerial Decision on measures against the spread of COVID-19 in specific third country national reception centers”, OJ 1584/2020, extended several times.

30 See Refugee Support Aegean, Foundation PRO ASYL, Communication to the Committee of Ministers of the CoE concerning the cases of *MSS v Belgium and Greece* and *RAHIMI v Greece* (Applications No 30696/09, 8687/08), Doc No DH-DD(2020)723, August 2020, para 61.

31 See HIAS Greece, Communication to the Committee of Ministers of the CoE concerning the cases of *MSS v Belgium and Greece* and *RAHIMI v Greece* (Applications No. 30696/09, 8687/08) DH-DD(2020)716, 15–16.

32 ibid.

33 See Ministry of Health, Circular on “measures to prevent the spread of coronavirus SARS-CoV-2 in reception facilities for unaccompanied minors”, 3 April 2020, Prot. No. Δ1β/ΓΠ οικ 22792.

34 By vulnerability here we are referring to special needs due their status as children, not to heightened vulnerability epidemiologically.

35 See EU Charter of Fundamental Rights, Arts 1, 4.

36 For a brief overview of the “hotspot approach to migration management” and to “hotspots”, the colloquial name for first reception facilities, see European Parliamentary Research Service, *Hotspots at EU External Borders: State of Play*, 2020 <www.europarl.europa.eu/thinktank/en/document.html?reference=EPRS_BRI(2020)652090> (last accessed 7 October 2020). For a detailed list of the tasks to be performed at hotspots by different agencies see D Neville et al, *On the Frontline: The Hotspot Approach to Managing Migration* (European Parliament 2016) pp 27–29.

37 Greek Council for Refugees and Oxfam, *Lesbos COVID-19 briefing: Update on the EU “hotspot” Moria by the Greek Council for Refugees and Oxfam,* 1 April 2020 <www.gcr.gr/en/news/press-releases-announcements/item/1420-oxfam-gcr-briefing-for-lesvos-amidst-the-coronavirus> (last accessed 7 October 2020).

38 See European Commission, COVID-19: Guidance on the implementation of relevant EU provisions in the area of asylum and return procedures and on resettlement (2020/C 126/02) OJ C 126/12.

39 European Parliament, “MEPs call for solidarity and measures to prevent Covid19 crisis in refugee camps” (News European Parliament, 3 April 2020) <www.europarl.europa.eu/news/en/headlines/world/20200402STO76413/meps-call-for-measures-to-prevent-covid19-crisis-in-refugee-camps> (last accessed 7 October 2020).

40 ActionAid Hellas et al, “Immediately honour commitments to relocate unaccompanied children from the Greek islands”, 3 April 2020 <reliefweb.int/sites/reliefweb.int/files/resources/Open%20Letter_Renew%20efforts%20to%20relocate%20unaccompanied%20children%20April%202020%20FINAL%20V.pdf> (last accessed 7 October 2020).

41 FRA, Relocation of unaccompanied children from Greece, 17 March 2020.

42 See information on the case in Refugee Support Aegean*, Evacuation of overcrowded island camps a legal imperative*, 21 April 2020 <rsaegean.org/en/evacuation-of-overcrowded-island-camps-a-legal-imperative/> (last accessed 7 October 2020).

43 Instead, the EU organized small-scale, ad hoc relocation. See analysis in E Tsourdi, “COVID-19, asylum in the EU, and the great expectations of solidarity” (2020) 32 International Journal of Refugee Law 374.

44 Greece has neither signed nor ratified Additional Protocol 4 to the ECHR, which establishes in its Art 2 the right to freedom of movement.

45 See ICCPR, Art 12(3).

46 See below, subs 4.

47 General Secretariat for Civil Protection, “Personal Data Policy”, s 4 <forma.gov.gr/docs/data-protection-policy.pdf> (in Greek) (no longer accessible).

48 For concerns see Homo Digitalis, “Open Letter” (30 March 2020) <www.homodigitalis.gr/wp-content/uploads/2020/04/%ce%95%CF%80%ce%B9%CF%83%CF%84%ce%BF%ce%BB%ce%AE_%ce%A0%ce%BF%ce%BB%ce%A0%ce%94_13033_HD_30.03.2020.pdf> (in Greek) (last accessed 7 October 2020).

49 This is because one of the exceptions was to go to the pharmacy or the doctor, which could reveal information about the health status of the individual.

50 For an analysis on tracking apps in the EU see H van Kolfschooten and A de Ruijter, “COVID-19 and Privacy in the European Union: A Legal Perspective on Contact Tracing” (2020) 41(3) Contemporary Security Policy 478; for a comparative study, see EU Agency for Fundamental Rights, “Coronavirus Pandemic in the EU – Fundamental Rights Implications: With a Focus on Contact-Tracing Apps” (April 2020).

51 A Kassimi, “Iχνηλάτηση τοΥ Iού Mέσω Kινητού” (“Virus Tracking via a Mobile Phone”) (*Kathimerini*, 21 June 2020) <www.kathimerini.gr/1083679/article/epikairothta/ellada/ixnhlathsh-toy-ioy-mesw-kinhtoy> (last accessed 7 October 2020).

52 Act of Legislative Content, “Emergency measures in response to the need to limit the dispersion of the coronavirus COVID-19”, note 3.

53 Art 5(1).

54 ibid.

55 Art 5(2).

56 Act of Legislative Content, “Measures to tackle coronavirus COVID-19 pandemic and other urgent provisions”, note 3.

57 Decision 2650/10.04.2020, “Settlement of more specific technical issues for the operation of the National Patient Register for COVID-19, in accordance with the provisions of articles twenty-nine of the Act of 30.3.2020 of the Legislative Content Act (PNP) ‘Measures to deal with the pandemic of the corona COVID-19 and other urgent provisions’” (A’ 75) and 83 of Law 4600/2019 (A’ 43)’ OG B’ 1298/10.4.2020 <www.e-nomothesia.gr/kat-ygeia/astheneies/koine-upourgike-apophase-2650-020.html> (in Greek) (last accessed 7 October 2020).

58 For criticism see Homo Digitalis, “COVID-19 και Ψηφιακά Δικαιώματα στην ελλάδα” (COVID-19 and Digital Rights in Greece) (22 April 2020) <www.homodigitalis.gr/wp-content/uploads/2020/04/HomoDigitalis_Report_COVID19_and_Digital_Rights_in_Greece_22.04.2020_Final.pdf> (last accessed 7 October 2020).

59 *S and Marper v UK* (2009) 48 EHRR 50, paras 107–21.

60 Joined Cases C-293/12 and C-594/12 *Digital Rights Ireland Ltd v Minister for Communications, Marine and Natural Resources and Others* (C-293/12) and *Kärntner Landesregierung and Others* (C-594/12) [2014] ECLI:EU:C:2014:238, para 64.

61 See Regulation (EU) 2016/679 of the European Parliament and of the Council of 27 April 2016 on the protection of natural persons with regard to the processing of personal data and on the free movement of such data, and repealing Directive 95/46/EC [2016] OJ L119/1, Art 9.

62 EODY, “Handling COVID-19 patients’ contacts” <eody.gov.gr/wp-content/uploads/2020/03/covid-19-diaxeirisi-epafon.pdf> (in Greek) (last accessed 7 October 2020).

63 Greek Data Protection Authority, “Guidelines on Processing of Personal Data in the Context of the Management of COVID-19” (18 March 2020) <www.dpa.gr/pls/portal/docs/PAGE/APDPX/ENGLISH_INDEX/NEWS/FILES/HELLENIC%20DPA_GUIDELINES_PROCESSING%20OF%20PERSONAL%20DATA_COVID-19.PDF> (in English) (last accessed 7 October 2020).

64 Art 57(3)(b).

65 For a list of guidelines issued by the Hellenic DPA see Hellenic DPA, “Guidelines of the Authority” <www.dpa.gr/portal/page?_pageid=33,120908&_dad=portal&_schema=PORTAL> (in Greek) (last accessed 7 October 2020).

66 ibid, para 1.

67 ibid, para 2.

68 Specifically, Arts 6(1)(c), 6(1)(d), 6(1)(e) as well as 9(2)(b), 9(2)(e), 9(2)(h) and 9(2)(i) of the GDPR.

69 DPA, note 63, para 4.

70 ibid, para 6.

71 Only the Greek and Belgian DPAs have adopted such approach. For a comparative overview see E Pappa, “Kορωνοϊός, Θερμομέτρηση και Προστασία Προσωπικών Δεδομένων” (Coronavirus, Temperature Checks and Protection of Personal Data) (*Lawspot*, 16 May 2020) <www.lawspot.gr/nomika-blogs/evelina_pappa/koronoios-thermometrisi-kai-prostasia-prosopikon-dedomenon> (in Greek) (last accessed 7 October 2020).

72 DPA, note 63, para 9.

73 Government Decree on “suspension of the submission of asylum applications”, OG A’ 45/2.3.2020 <bit.ly/2XFsMvb> (in Greek) (last accessed 7 October 2020).

74 See instead, “UNHCR statement on the situation at the Turkey-EU border” (UNHCR, 2 March 2020) <bit.ly/3bhsM8D> (last accessed 7 October 2020).

75 See analysis in Refugee Support Aegean, “Rights denied during Greek asylum procedure suspension”, April 2020.

76 See Directive 2013/33/EU of the European Parliament and of the Council of 26 June 2013 laying down standards for the reception of applicants for international protection (Reception Conditions Directive), OJ L 180/96, Arts 8–11 and analysis in E Tsourdi, “Asylum Detention in EU Law: Falling between Two Stools?” (2016) 35(1) Refugee Survey Quarterly 7 and E Tsourdi, “International human rights law, EU law, and alternatives to immigration detention: shaping control standards and judicial interaction in an heterarchy”, in M Moraru et al (eds), *Law and Judicial Dialogue on the Return of Irregular Migrants from the European Union* (Hart Publishing 2020) pp 167–91.

77 Joint ministerial decision of the Minister of Health and the Minister of Interior, Migration and Asylum concerning the temporary suspension of receiving public according to the provisions of Art 15 of the Act of Legislative Content issued on 15 March 2020, No 12687/2020 (B’ 936).

78 Joint ministerial decision of the Minister of Health and the Minister of Interior, Migration and Asylum concerning the extension of application of joint ministerial decision 12687/16.3.2020 on the temporary suspension of receiving public (B’ 939), OJ 3332/2020.

79 See ECRE, Information Sheet, 28 May 2020: Covid-19 Measures Related to Asylum and Migration Across Europe, ECRE 2020, 5.

80 Eg Regulation (EU) 2016/399 of the European Parliament and of the Council of 9 March 2016 on a Union Code on the rules governing the movement of persons across borders (Schengen Borders Code), OJ L 77/1, Arts 3(b) and 7 and EU Charter of Fundamental Rights, Arts 18 and 19 and analysis in FRA and Council of Europe, *Fundamental Rights of refugees, asylum applicants and migrants at the European borders*, 2020.

81 See UNHCR, “Practical Recommendations and Good Practice to Address Protection Concerns in the Context of the COVID-19 Pandemic”, April 2020.

82 Section III.1.b.

83 See FRA, Coronavirus Pandemic in the EU-Fundamental Rights Implications: Bulletin No 1, April 2020, 24.

84 The document bears the Protocol Number 12540/2020.

85 The document bears the Protocol Number 13517/2020.

86 Joint Ministerial Decision of Ministers of Citizens’ Protection, Health and Interior No Δ1α/Γ.Π.οικ.20036/22.3.2020 (B'986) concerning the temporary restriction of movement to contain the danger of spreading coronavirus) OG B’ 1272/9.4.2020.

87 The Greek Government doubled the amount of the fines for the period 18–20 April to prevent unnecessary movement during the Orthodox Easter holiday. An additional penalty of removal of the driving licence was imposed on drivers in breach of the restrictions. Ministries of Citizen Protection, Health and Interior, Joint Ministerial Decision, “Penalties in case of violation of the measures to restrict the movement of citizens to deal with the risk of dispersion of the coronavirus COVID-19 during the period from 18.4.2020 to 20.4.2020”, OG B’ 1477/17-4-2020 <www.dsanet.gr/Epikairothta/Nomothesia/YA%2025771_1477.htm> (in Greek) (last accessed 7 October 2020).

88 The Explanatory Memorandum of the updated Penal Code notes that Art 285 was modified to combine previous Arts 283 and 284 and includes the aggravated form of the offence. See Draft Legislation “Ratification of the Penal Code”, Explanatory Memorandum, pp 55–56 <www.hellenicparliament.gr/UserFiles/2f026f42-950c-4efc-b950-340c4fb76a24/k-poinkod-eis-NEO.pdf> (in Greek) (last accessed 7 October 2020).

89 For an analysis see F Spyropoulos, “H Διάδοση τοΥ Iού COVID-19 Υπό το Πρίσμα τοΥ Aρ. 285 ΠK” (The Transmission of COVID-19 Through the Lens of Article 285 of the Penal Code) (Crime Times, April 2020) <www.crimetimes.gr/h-%ce%B4%ce%B9%ce%AC%ce%B4%ce%BF%CF%83%ce%B7-%CF%84%ce%BF%CF%85-%ce%B9%ce%BF%CF%8D-covid-19-%CF%85%CF%80%CF%8C-%CF%84%ce%BF-%CF%80%CF%81%ce%AF%CF%83%ce%bc%ce%B1-%CF%84%ce%BF%CF%85-%ce%B1%CF%81-285/> (in Greek) (last accessed 7 October 2020).

90 Greek Penal Code, Art 285(4).

91 Few circulars were issued concerning the postponement of court proceedings.

92 Office of the Public Prosecutor at the Court of Cassation (Άρειος Πάγος), “Circular No 4/2020” (12 March 2020) <eisap.gr/εγκύκλιος-04-2020/> (in Greek) (last accessed 7 October 2020).

93 Office of the Public Prosecutor at the Court of Cassation (Άρειος Πάγος), “Circular No 7/2020” (31 March 2020) <eisap.gr/εγκύκλιος-07-2020/> (in Greek) (last accessed 7 October 2020).

94 See Greek Constitution, Art 21(3).

95 See Greek Constitution, Art 25(4).

96 Art 285 requires *in concreto* danger. See Spyropoulos, supra, note 89.

97 However, on 22 September 2020, the Office of the Public Prosecutor at the Court of Cassation issued another circular in which the Public Prosecutor referred to the phenomenon of individuals who refused to abide by the rules concerning the use of masks in specified spaces. The Prosecutor further noted the establishment of a sui generis state of emergency and pointed out that on top of Art 285 of the Greek Penal Code, Art 183 of the same Code, which concerns procurement to disobedience, could also be invoked in respect of such individuals who refuse to wear masks and socially distance. The circular further includes an interpretation of Art 183 of the Greek Penal Code in accordance with academic literature as guidance for prosecutors. See Office of the Public Prosecutor at the Court of Cassation (Άρειος Πάγος), “Circular No 16/2020” (22 September 2020) <eisap.gr/εγκύκλιος-16-2020/> (last accessed 7 October 2020).

98 “Kλινική ‘Tαξιάρχαι’: Ποινική Δίωξη σε Bαθμό KακοΥργήματος κατά ΥπεΥθύνων και Γιατρών” (Prosecution for Felony against Managers and Doctors) (*Kathimerini*, 31 July 2020) <www.kathimerini.gr/1090289/article/epikairothta/ellada/klinikh-ta3iarxai-poinikh-diw3h-se-va8mo-kakoyrghmatos-kata-ypey8ynwn-kai-giatrwn> (in Greek) (last accessed 7 October 2020).

99 O Ştefan, “COVID-19 Soft Law: Voluminous, Effective, Legitimate? A Research Agenda”, European Forum of European Papers (3 June 2020) 1–2.

